# The correlation of sulfonation reaction kinetics with the degree of sulfonation (DS) and its effects on microstructure and morphology of electrospun fibers for the membrane of fuel cells

**DOI:** 10.1039/d2ra05587b

**Published:** 2023-01-17

**Authors:** Ronaldo P. Parreño

**Affiliations:** a Chemicals and Energy Division, Industrial Technology Development Institute (ITDI), Department of Science and Technology (DOST) Taguig 1631 Philippines rpparreno@itdi.dost.gov.ph ronaldo_parrenojr@dlsu.edu.ph; b Department of Chemical Engineering, De La Salle University 2401 Taft Avenue Manila 1004 Philippines

## Abstract

Sulfonation is among the frequently used surface modification methods to endow new surface properties on polymer fibers. Direct sulfonation is the simplest chemical modification *via* incorporation of sulfonate functional groups onto fibers' surfaces at ordinary conditions. However, the process of functionalization compromises other properties of the electrospun membrane due to the extent of sulfonation reaction that occurred. This study was carried out to determine the effects on microstructure and morphology of crosslinked electrospun polybenzoxazine (PBz) fibers by correlating the reaction kinetics using a specific reaction parameter with the degree of sulfonation (DS). The kinetics of sulfonation reaction for PBz fibers is an electrophilic-based, first-type substitution mechanism where only one sulfonic acid (–SO_3_H) group is attached to each of the repeating units in the aromatic structure of PBz under ordinary conditions. From a previous study, the rate of change of ion exchange capacity (IEC) was derived as a logarithmic correlation that increased rapidly for some time and became constant. This study employed the derived empirical relationship based on reaction kinetics to correlate the degree of sulfonation (DS) with IEC at varying reaction time. DS was calculated as 55%, 66% and 77%, for 3, 6 and 24 h, respectively. The maximum IEC of 2.71 with the corresponding DS of 100% was obtained theoretically but since sulfonation reaction kinetics at ordinary conditions proceeds at slower phase, it can only attain 86% DS with maximum IEC of 2.44 at a calculated time of 48 h. Sulfonation was confirmed by the functional groups present in the structural composition of PBz fibers using ATR-FTIR which were quantified based on the absorbance in the IR spectra showing an increased peak height with increased reaction time for the first 6 h. Morphological analyses using SEM images revealed an increase in fiber diameter with increased sulfonation time. In addition, the higher reaction time showed the effects of longer acid exposure which compromised the fibers' structural integrity with the presence of broken fibers and defects on fibers' surfaces after 24 h. The electrospun PBz fibers sulfonated for 6 h achieved the highest IEC value with DS of 66% which was enough to attain the balance of degree of sulfonation with electrochemical and morphological properties.

## Abstract 2

Sulfonation reaction on electrospun fibers with the effect of degree of sulfonation on fibers' microstructure and morphology.

## Graphical abstract

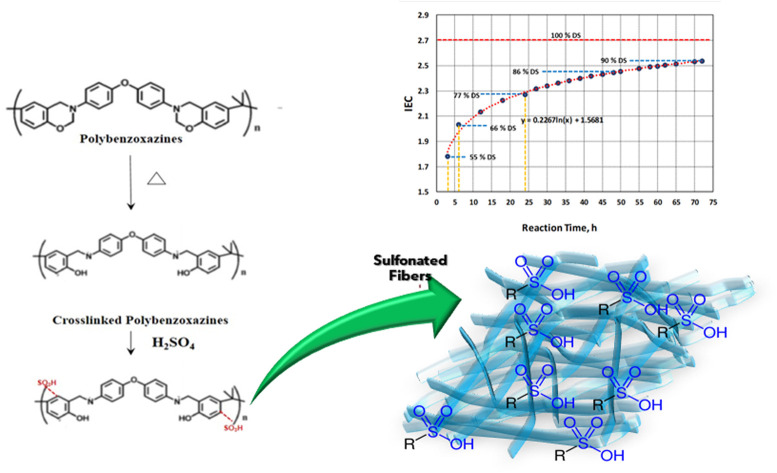

## 1. Introduction

In the advancement of polymeric materials for high-performance applications, functionalization by physical and chemical modifications is now extensively pursued as an effective way to enhance the functional properties for better and superior materials. It has been proven that one single material does not exhibit all the desired properties, thus modification may result in improving its properties.^1^ Polymers are classified among the advanced materials with increasing integral function and variety as well as high added-values.^2^ Their performance for various applications depends mainly on the bulk properties in combination with the surface properties but more often polymers do not have the needed surface properties for target applications.^3^ Due to this constraint, many surface modification methods are explored for polymeric materials such as coating, blending, compositing, chemical, grafting or a combination of these methods.^3^

Sulfonation is one of the simplest approaches frequently used for modification of polymer membrane to provide additional functional properties and improve the hydrophilicity for higher water flux with the enhancement of antifouling and hydration capacities.^1^ But recent studies examined other functional properties obtained after surface modification which have effects on the overall performance of the membrane such as microstructure, mechanical and thermal properties, and more recently, proton conductivity enhancement for fuel cell application.^4^ The simplest sulfonation process for polymeric materials is by direct sulfonation using concentrated sulfuric acid (H_2_SO_4_) as the sulfonating agent conducted under ordinary condition.^1^

In our previous work, direct sulfonation process was employed in investigating the possible effects of using concentrated H_2_SO_4_ to functionalize thermally-crosslinked electrospun PBz fibers for membrane of fuel cells. First consideration in the study was to know the suitability of the concentrated acid as sulfonating agent for this type of thermoset resin polymeric material. It is of utmost importance that the integrity of the electrospun fibers during the sulfonation reaction remained intact in its original physical form as fiber mat to make it suitable for membrane of fuel cells. After undergoing sulfonation reaction, the desired properties endowed by functionalization were evaluated as evidence of successful sulfonation. Ion exchange capacity (IEC) was determined as a function of sulfonation reaction time which was an indicator of electrochemical capacity for its intended application as membrane of fuel cells.^5^ The empirical model formulated based on the experimental data showed statistically significant correlation which could be further utilized to describe relevant aspects of microstructure and morphology of sulfonated PBz fibers. Thus, this study looked into the effects of degree of sulfonation (DS) on microstructure and morphology of the electrospun PBz fibers by correlating it with sulfonation reaction kinetics. After successfully understanding the underlying concept behind the sulfonation reaction mechanism and examining the reaction parameters of the sulfonation kinetics for PBz as polymer of interest, it would be of great benefit to understand the extent of sulfonation reaction in terms of reaction degree. The relation between the reaction time and IEC value was used for extracting the DS which was used as basis for the changes observed in microstructure and morphology after the sulfonation reaction.

## 2. Experimental

### 2.1 Materials

Polybenzoxazines (PBz) was prepared in the lab as reported in the work of Lin *et al.*^6^ Electrospun polybenzoxazines (PBz) fiber mats were prepared using vertical set-up electrospinning process based on the procedure described in the work of Parreño *et al.*^5^ Concentrated H_2_SO_4_ (analytical grade, 96–97%) was purchased from Aencore and used as received. Dimethylsulfoxide (DMSO) (ACS grade, Echo, 99.9%), and tetrahydrofuran (THF) (inhibitor free high purity, Tedia, 99.8%) were also used as received.

### 2.2 Direct sulfonation

Direct sulfonation process of electrospun fiber mat sample was conducted according to the procedure described in the work of Esmaielzadeh and Ahmadizadegan.^7^ Fiber mat samples were prepared (approx. 1 cm × 1 cm) from the crosslinked electrospun PBz fibers. Then, the samples were immersed in concentrated H_2_SO_4_ at room temperature for desired reaction time of 3 h, 6 h and 24 h. After reaction took place, the samples were removed from sulfuric acid and washed with deionized water repeatedly, to remove the residual sulfuric acid until the pH of wash water was greater than 5. Additional post-washing treatment was applied to the mat samples after sulfonation as reported from literature.^1^ In acetone/water (1/1) (v/v), the sulfonated mat samples were immersed for 5 min. Then, samples were removed from the acetone/water mixture and immersed in pure acetone for 10 min. Subsequently, the samples were dried in the oven (Deng Yng, DH 400) at temperature of 50 °C for 24 h. The samples were stored for further characterization and correlation study.

### 2.3 Ion exchange capacity (IEC)

The IEC was determined using modified back titration procedure described in the work of Huang *et al.*^1^ Prior to back titration procedure, fiber mat samples were prepared by neutralization in 0.01 M sodium hydroxide aqueous solution at sample to NaOH ratio of 0.025 g/10 mL for 72 h. This fully converted the sulfonated mat samples into its sodium salt form. Then, dilute sulfuric acid with concentration of 0.003 M was employed to back titrate the NaOH aqueous solution that was partially neutralized by the sulfonated mat samples. The neutral point in the back titration was predicted by using universal indicator. The volume of the sulfuric acid used in the titration was used for obtaining the IEC of the sample.

### 2.4 Degree of sulfonation (DS)

The degree of sulfonation (DS) was determined using the equation based on the work of Huang *et al.*^1^1
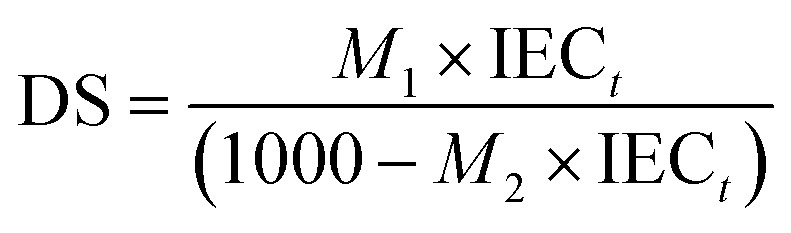
where DS is the degree of sulfonation, *M*_1_ (236 Dalton) is the molecular weight of the sulfonated PBz fibers, *M*_2_ (133 Dalton) is the molecular weight of the pristine PBz fibers, and IEC is the ion exchange capacity obtained at reaction time (*t*) from the works of Parreno *et al.*^5^

### 2.5 Characterization of sulfonated electrospun fibers

The electrospun PBz fibers were characterized using attenuated total reflectance (ATR) with Fourier-transform infrared (FTIR) spectrometer (PerkinElmer, MA, USA) at the wavenumbers between 400 cm^−1^ to 4000 cm^−1^ to confirm the sulfonation reaction. Changes in the microstructure and morphology were analyzed based on the effect of degree of sulfonation (DS) by using scanning electron microscope (SEM) (FEI Helioz Nanolab 600i, Eindhoven, The Netherlands) at the Advanced Device and Materials Testing Laboratory (ADMATEL) of DOST.

## 3. Results and discussions

### 3.1 Sulfonation reaction kinetics

The reaction mechanism for direct sulfonation of electrospun PBz fibers was determined as electrophilic first-type substitution based on the results of sulfonation reaction using concentrated H_2_SO_4_ as sulfonating agent at ordinary condition. The reaction occurred with only one sulfonic acid (–SO_3_H) group attached preferentially to the *ortho*-position of the aromatic ring by electrophilic substitutions.^1,7^ According to Donaldson and Wren,^8^ the basic principle of reaction kinetics involves the rate of transformation of chemical compounds from reactant species into products relating the chemical reaction that occurred to the rate coefficients, reaction order, rate expression, reaction mechanisms and steady-state concentration. From previous study, the rate parameters (IEC *vs.* reaction time) were extracted for first-order type electrophilic substitution mechanism of sulfonic acid (–SO_3_H) in the aromatic ring of PBz.^5^ Further analyzing the sulfonation reaction, according to Ma *et al.*,^9^ the reactive characteristic time (*t*_½_) is:

2 *t*_½_ = ln 2/*k*

For phenol sulfonation by H_2_SO_4_, the value of rate constant (*k*) of about 103–104 L mol^−1^ s^−1^ was based on reported data from the work of Cerfontain *et al.*^10^ This was used for estimation of the reactive time, *t*_½_ which was computed at about 0.01–0.1 m s^−1^.^9^ Thus, at ordinary condition a slower reactive characteristic time occurs which was validated during the actual sulfonation reaction conducted for PBz fibers. The reaction proceeds at a slower phase due to process conditions related to the energy barrier and yet it was effective in attaining the highest rate of reaction at 24 h reaction time.

Sulfonation reaction as functionalization for membrane provides important properties for polymer electrolyte of fuel cells. One of the properties is the ion exchange capacity (IEC) defined as the number of sulfonate groups in the polymer which influences its electrochemical capacity for proton transport. However, it is not enough to obtain high IEC values to make it suitable for polymer electrolyte membrane. The effects of degree of sulfonation (DS) on microstructure and morphological property of the polymer should also be taken into considerations and evaluated.

In our previous work, the reaction kinetics model was established for sulfonation of thermally-crosslinked PBz fibers with concentrated H_2_SO_4_ at ordinary condition. The results showed reaction time has direct correlation with rate of change of IEC while other process conditions such as reaction temperature and acid to polymer fiber ratio were maintained constant. According to Ma *et al.*,^9^ the other operating conditions in sulfonation process are reaction temperature, acid feed rate, stirring speed and ageing time which were not considered at ordinary condition to simplify the model. These other parameters could be investigated for determining optimal operating conditions of the sulfonation process at extra ordinary condition that could speed up the reaction process by applying heat. The reaction kinetics based on the correlation of the reaction time with rate of change of IEC exhibited a non-linear relationship where the IEC increased logarithmically with increased reaction time.^5^ The IEC values at different reaction times are summarized in Table 1. The IEC values for 3 h, 6 h and 24 h reaction times were computed from actual experimental results while the IEC values for 48 h and 72 h reaction times were calculated using the empirical model,^5^

3 *y*(IEC) = 0.2267 ln *x*(*t*) + 1.5681

where *y* is the IEC value and *x* is the reaction time, *t* with a very high correlation coefficient of 0.9797.

**Table 1:** IEC values at different reaction time

Reaction time, h	IEC, meq. g^−1^
3	1.78
6	2.03
24	2.27
48	2.44^a^
72	2.54^a^

a Based on the empirical model.

The use of experimental data from previous work to come up with a valid model was used in predicting IEC values using reaction time (48 h and 72 h) as the predictor variable. The limited resources available for this study gave an opportunity of using empirical research to provide additional insights into the research investigation of the correlation of sulfonation reaction kinetics with degree of sulfonation which was a valid option that is proven to work and acceptable.

**Fig. 1:**
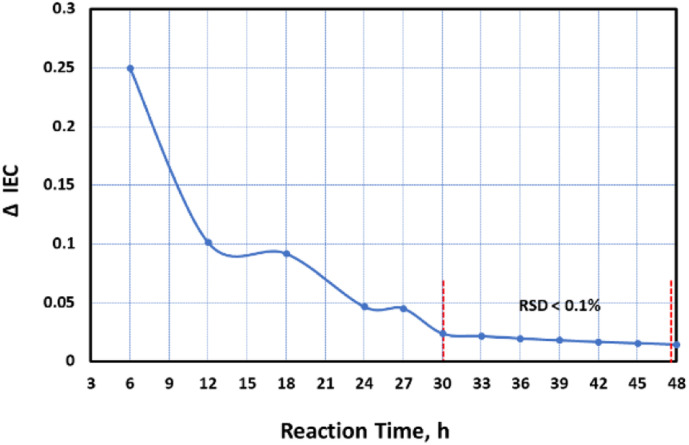
Rate of change in IEC value over time.

The rate of change in the IEC (ΔIEC) with reaction time were also analyzed based on the experimental values as well as the ΔIEC values after 24 h based on the empirical model. The results shown in Fig. 1 revealed that the rate of change over time reached its highest at 3 h with maximum value of IEC at 27 h. But the rate of change starting from 30 h was statistically insignificant having very small mean difference with relative standard deviation (RSD) of less than 0.1%.

### 3.2 Correlation of reaction kinetics with degree of sulfonation

Degree of sulfonation (DS) is a vital indicator of effective sulfonation reaction with the introduction of sulfonic acid groups onto the polymer fibers' surfaces. From previous work the reaction kinetics in relation to the change in IEC was investigated but without looking into the adverse effects on PBz fibers' microstructure and morphology. According to Banerjee and Kar,^4^ the degree of sulfonation (DS) brings about enhancement in the proton conductivity due to the hydrophilic domains present in the polymer matrix but on the other hand, a very high DS affects the mechanical properties of the polymer. Although the stability of PBz fibers in relation to its thermal resistance was not drastically affected, the start of degradation changed with an early onset temperature for sulfonated sample at 24 h.^5^ Polymers like PBz with considerable amount of oxygen functional groups in the surface such as epoxy, hydroxyl, and carboxylic acid groups can easily decompose at about 200 °C.^11^ But the changes in microstructure and morphology were not analyzed based on the extent of sulfonation reaction.

Degree of sulfonation (DS) was determined based on reaction time of 3 h, 6 h and 24 h using eqn (1) with IEC values of 1.78, 2.03 and 2.27, respectively. Reaction time is one of the important factors of direct sulfonation along with operating temperature and sulfonating agent which contribute to the desired DS.^12^ The incorporation of sulfonic acids to the PBz fibers attained 55% DS after 3 h of reaction time and increased by 11% after 6 h and 24 h with DS of 66% and 77%, respectively as shown in Fig. 3. However, as indicated in the IEC values which reached a plateau between 18 and 24 h, the increase in DS between 6 h to 24 h which span 18 h of reaction time resulted to only an incremental change in DS of 11%. This confirmed the slower phase of reaction at ordinary condition of sulfonation process. Although it was a slow process, it already attained the DS above 60% after 6 h which is the extent of sulfonation (60–80% DS) required to obtain a high level of proton conductivity and structural strength.^13^

**Fig. 2:**
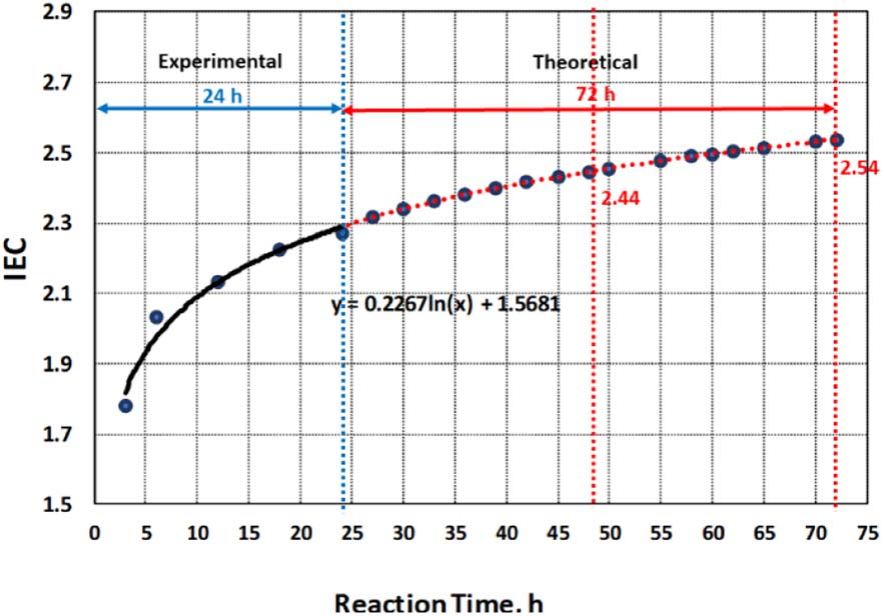
Predicted values of IEC at 48 h and 72 h of sulfonation.

**Fig. 3:**
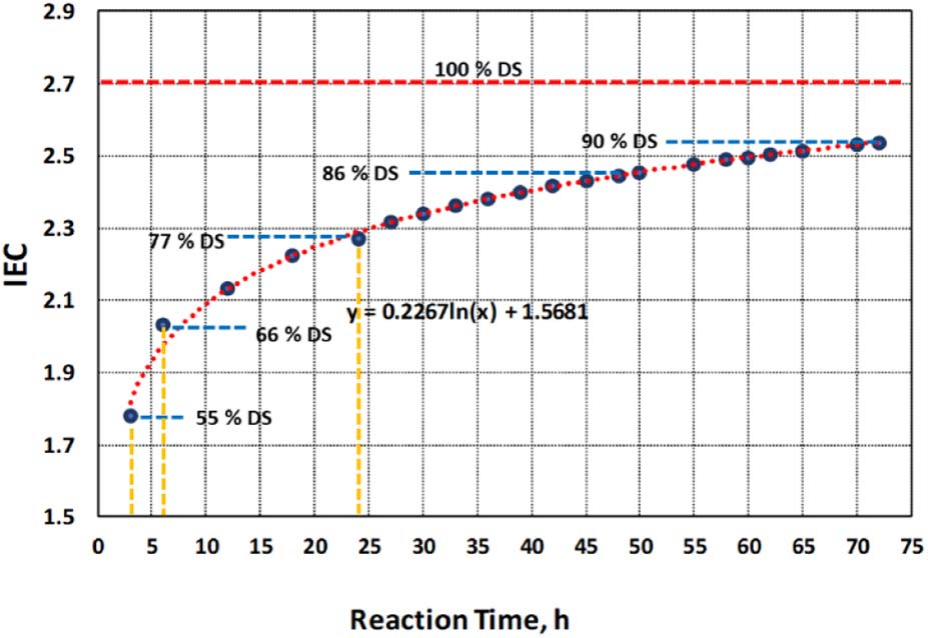
Correlation of DS with the rate of change of IEC over time at 48 h and 72 h.

The correlation of DS with IEC and its effects on the microstructure and morphology were further analyzed beyond the experimental value of 2.27 at reaction time of 24 h. This is in accordance with the recommended DS range of 60–80% which has direct influence on the structure and morphology of the polymer membrane.^4^ Based on the model obtained from the experimental data, the predicted values of IEC after 48 h and 72 h of sulfonation process were obtained as shown in Fig. 2. By correlating the IEC values for each reaction time to the extent of sulfonation process of PBz fibers, the DS were also theoretically calculated from the graph in Fig. 3.

At 100% DS, the computed theoretical value of IEC is 2.71 which is the maximum IEC for direct sulfonation process of PBz fibers with concentrated H_2_SO_4_ at ordinary condition. The DS attained 86% and 90% DS after 48 h and 72 h, respectively. The recommended high value limit of 80% DS was attained at approximately 30 h reaction time. But based on actual experimental run of sulfonation reaction at 72 h reaction time, the process was not possible since it affected the stability of the fiber mat which formed into gel during sulfonation.

### 3.3 Effects of degree of sulfonation on microstructure and morphology

**Fig. 4:**
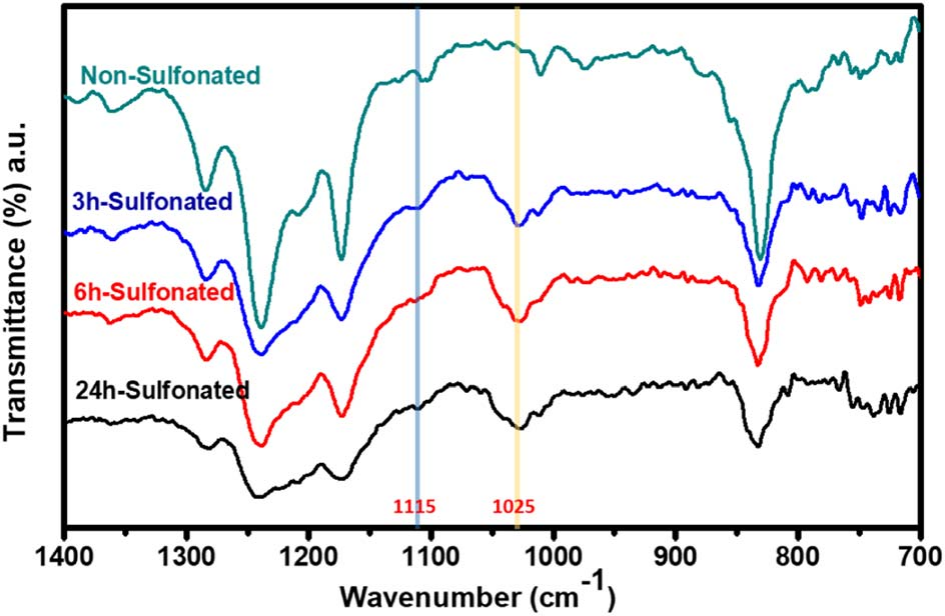
Structural evolution of sulfonated PBz fibers.

The change in structural compositions of the PBz fibers was directly measured using attenuated total reflectance (ATR) with Fourier transform infrared (FTIR) spectroscopy. The characteristic peaks at wavenumbers of 1115 and 1025 cm^−1^ were assigned to the symmetric and asymmetric O=S=O stretching vibrations, respectively, which are excellent evidence of the presence of sulfonic acid groups after sulfonation onto fibers' surfaces as shown in Fig. 4. These results proved that all the three samples that undergone sulfonation process at different reaction time exhibited new peaks. The sulfonic acid groups were very evident in the fibers which were in agreement with the studies of Taek *et al.*^14^ and Tabekh *et al.*^15^

Further analyses of the peaks and its height by applying baseline correction of absorbance and integration of peaks present in the IR spectra, the height of the peak for the sulfonate groups associated with more prominent asymmetric stretching vibration could be quantified as shown in Table 2. Comparing the height of the peak associated with sulfonate functional groups at peak of 1025 cm^−1^ present in the three samples, it revealed that the longer the reaction time, the higher the height of the peak. It also verified the effect of longer reaction time of sulfonation process using concentrated H_2_SO_4_ at low temperature which proceeded at faster phase with more presence of functional groups within 6 h. Then, the sulfonation kinetics reached a plateau with slightly lower rate of sulfonation with slightly lower peak height attributed to aggregation over time which eventually damaged the surfaces of the fibers at reaction time of 24 h.

**Table 2:** Peak height of sulfonate groups in the sulfonated PBz fibers

Sample	Reaction time (h)	Peak (cm^−1^)	Peak height
Non-sulfonated	0	None	0.00
3 h-Sulfonated	3	1025	0.87
6 h-Sulfonated	6	1025	0.96
24 h-Sulfonated	24	1025	0.79

**Fig. 5:**
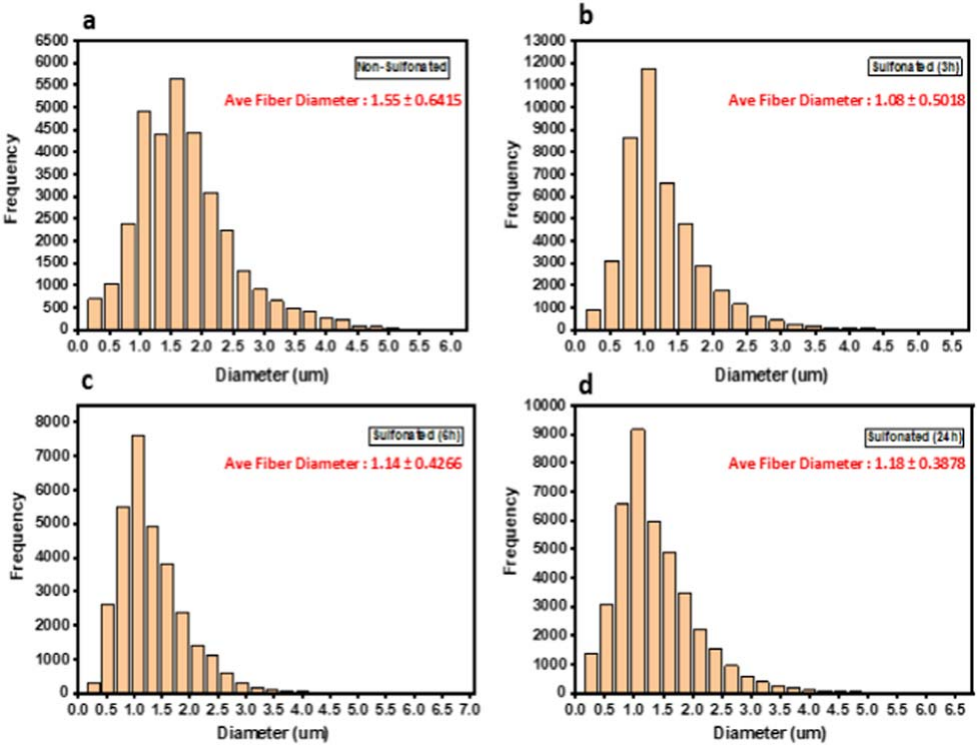
Fiber diameter and distribution of sulfonated PBz fibers.

Morphological analyses were carried out by using scanning electron microscopy (SEM) to reveal changes in the diameters of the fibers. Based on the SEM images, it revealed that the diameter of fibers increased with increasing sulfonation time. The non-sulfonated electrospun PBz fibers showed a uniform fiber morphology with an average fiber diameter of 1.55 ± 0.6415 μm and distribution of fiber diameter concentrated between 0.5–3.0 μm as shown in Fig. 5a. For the sulfonated fibers, the average fiber diameter for all samples decreased slightly from 1.08 ± 0.5018 μm for 3 h, 1.14 ± 0.4266 μm for 6 h, and 1.18 ± 0.3878 μm for 24 h as shown in Fig. 5b–d.

In the study of Yao *et al.*^16^ and Todsapon,^17^ sulfonation using concentrated H_2_SO_4_ (98%) caused the dissolution of electrospun fibers which requires post-treatment to crosslink and improve fiber resistance to concentrated acid. This is contrary to the works of Jalal *et al.*,^18^ where the fiber diameter gradually increases with increased sulfonation reaction time but using diluted sulfuric acid. However, this study showed that fiber diameter decreased compared with non-sulfonated fibers by 23–30% which was observed similar to the results of previous works.^16,17^ Comparing only the sulfonated fibers with increasing sulfonation reaction time, the fibers slightly increased from 1.08 to 1.18. This observation could be attributed to the amount of sulfonate groups that adhered to the surfaces of the fibers with increasing time (3 h to 24 h) and were able to retain due to fiber resistance from crosslinked reaction of PBz. In terms of pore structure of the sulfonated fibers that showed increased fiber diameter due to sulfonate groups in the surfaces, the porosity will slightly decrease caused by the surface adherence of –SO_3_H which now occupied the internal spaces between these fibers after the modification.^19^

**Fig. 6:**
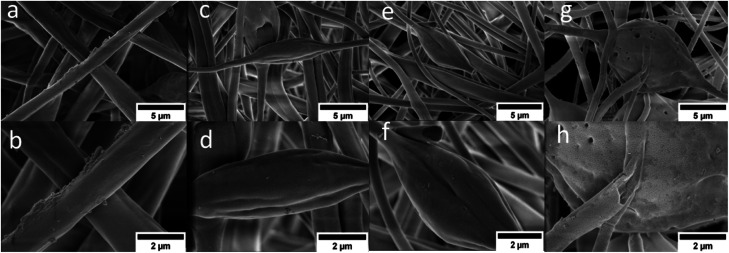
SEM images of (a) and (b) non-sulfonated, (c) and (d) sulfonated fibers at 3 h, (e) and (f) sulfonated fibers at 6 h and (g) and (h) sulfonated fibers at 24 h. (10 000 and 25 0000 magnification; scale: 5 and 2 μm).

The SEM images of the sulfonated fibers compared to pristine PBz at lower magnification 0f 10 000 and scale of 5 μm showed almost the same uniform random-oriented fiber morphology as shown in Fig. 6a–h. But looking closer at higher magnification of 25 000 and scale of 2 μm, the wetted fibers' surfaces with sulfonates were more evident due to the acid exposure. The higher the reaction time, the longer the acid exposure which showed in the SEM images in Fig. 6c–h where fibers' structural integrity were no longer intact with the presence of broken fibers and damaged surfaces. At the reaction time of 24 h, the DS attained was 77% with ion exchange capacity of 2.27. However, the effects of reaction time validated the extent of sulfonation degree even at slower phase which was able to achieve higher electrochemical property but affected the mechanical integrity of polymer fibers. The PBz fibers exposed to sulfonation for 24 h reaction time showed in its surface texture the presence of imperfections and defects on the fibers which could be attributed to the aggregates of sulfonic acid groups that caused the damage due to longer reaction time.^20^ This proved that sulfonation at 6 h with DS of 66% achieved the balance of electrochemical and morphological properties.

## 4. Conclusions

Sulfonation reaction at ordinary condition is one of the simplest approaches of functionalization of polymeric membrane to achieve one of the important indicators of electrochemical capacity for fuel cell applications. By investigating the correlation of reaction time and IEC value with the degree of sulfonation (DS), morphology as another important property for electrolyte membrane was determined in consideration of its performance. The results of this study proved that the sulfonation reaction kinetics played a significant role in determining the correlation of IEC with DS at increasing reaction time. Theoretical values from derived model compared to actual experimental results confirmed the threshold limit of reaction degree at 66% that surface modification can achieve in terms of electrochemical and morphological properties of polymer fibers for fuel cell applications. Thus, electrospun PBz fibers sulfonated for 6 h achieved the highest possible IEC value at DS of 66% which were enough to attain the balance of degree of sulfonation reaction with electrochemical and morphological properties.

## Author contributions

Conceptualization, R. P. P. J.; methodology, R. P. P. J.; formal analysis, R. P. P. J. investigation, R. P. P. J.; data curation, R. P. P. J.; resources, R. P. P. J.; writing—original draft preparation, R. P. P. J.; writing—review and editing, R. P. P. J.; funding acquisition, R. P. P. J.

## Conflicts of interest

There are no conflicts to declare.

## Supplementary Material
